# Prevalence and Molecular Identification of *Entamoeba* spp. in Non-human Primates in a Zoological Garden in Nanjing, China

**DOI:** 10.3389/fvets.2022.906822

**Published:** 2022-05-30

**Authors:** Xinchao Liu, Guangbin Bao, Menglong Yue, Yi Fang, Yueyue Gu, Wenchao Li, Youfang Gu, Wangkun Cheng, Mingmin Lu

**Affiliations:** ^1^Anhui Province Key Laboratory of Animal Nutritional Regulation and Health, College of Animal Science, Anhui Science and Technology University, Fengyang, China; ^2^Nanjing Hongshan Forest Zoo, Nanjing, China; ^3^MOE Joint International Research Laboratory of Animal Health and Food Safety, College of Veterinary Medicine, Nanjing Agricultural University, Nanjing, China

**Keywords:** molecular identification, non-human primates, zoological garden, *Entamoeba* spp., infection

## Abstract

**Objective:**

*Entamoeba* spp. are globally distributed zoonotic parasites that infect various hosts, among which non-human primates (NHPs) have been identified as one of the most common hosts of these parasites. Consequently, the infections of *Entamoeba* spp. in captive NHPs from Nanjing Hongshan Forest Zoo in China were investigated in order to assess their zoonotic potential.

**Methods:**

A total of 120 fresh fecal samples, including 19 species of NHPs, were collected from four breeding bases of the zoo from May to June 2019. The infections of six species of *Entamoeba* spp. were detected by PCR using the 16S or 18S rDNA-specific primers, and the positive samples were sequenced and analyzed.

**Results:**

*Entamoeba* spp. were detected as positive in 59 NHPs fecal samples (49.17%), including five *Entamoeba* species: *Entamoeba histolytica* (7.50%), *E. dispar* (22.50%), *E. coli* (22.50%), *E. chattoni* (10.00%) and *E. nuttalli* (1.67%). Infection with one *Entamoeba* species was more common (35%) than co-infections (13.33%) or infections with three *Entamoeba* species (0.83%). There was a significantly higher prevalence rate of *Entamoeba* spp. in the species *Pongo pygmaeus* and *Macaca mulatta* than in *Papio* sp., *Mandrillus sphinx*, and *Saimiri sciureus*.

**Conclusion:**

*Entamoeba* spp. are highly prevalent in the NHPs raised in Nanjing Hongshan Forest Zoo. Therefore, attention should be paid to the development of containment strategies of *Entamoeba* spp. in this zoological garden.

## Introduction

*Entamoeba histolytica*, the causative agent of amebiasis, is a protozoan parasite that infects about 10% of the world' population and causes 100,000 deaths per year globally (1, 2). The genus *Entamoeba* includes several species, such as *E. dispar* and *E. moshkovskii*, which are morphologically similar to *E. histolytica* (3). Although *E. dispar* and *E. moshkovskii* have no apparent invasive potential, these two species exhibit some pathogenicity (4, 5). *E. coli, E. hatmanni*, and *E. nuttalli* are considered non-pathogenic; however, those species are frequently found in the stools of several animals (6–8). As a reference method for parasitological diagnosis, the microscopic examination has been widely used to identify *Entamoeba* species, even though it lacks the ability to differentiate the infection caused by *E. histolytica* from non-pathogenic *Entamoeba* spp. (9). Therefore, molecular methods have been preferably used to identify and investigate the epidemiology of *Entamoeba* spp. in human and animal hosts (6, 7, 10, 11).

Among the hosts of *Entamoeba* spp., non-human primates (NHPs) have a close phylogenetic relationship with human, which increases the risk of *Entamoeba* spp. transmission between NHPs and humans. Previous studies showed that both captive and free-ranging NHPs are frequently infected with *Entamoeba* spp. (11, 12), and many *Entamoeba* spp. were identified in NHPs, especially *E. histolytica* (6, 13). Given that captive NHPs have more opportunities to contact with humans than free-ranging, they are a major concern group of host with a higher potential for zoonotic infections (6, 14).

Nanjing Hongshan Forest Zoo is one of the most distinctive zoological gardens in China with abundant NHPs animal resources and colorful theme activities, attracting millions of visitors every year. However, there is inadequate information on the epidemic of parasitic zoonoses within this zoological garden. The presence of zoonotic parasites poses a threat to the health of NHPs, which may in turn cause zoonotic transmission events between tourists and NHPs. Consequently, in this study, we aim to investigate the prevalence of *Entamoeba* spp. in NHPs in Nanjing Hongshan Forest Zoo to assess the potential for zoonotic transmission.

## Materials and Methods

### Collection of Stool Samples

From May to June 2019, stool samples of 120 NHPs were collected from four breeding bases, including the headquarter (26/120), Gaochun base (26/120), Jing'an base (64/120), and rescue center (4/120) of Nanjing Hongshan Forest Zoo. There were 19 species included in 120 NHPs. About 15*g* fecal sample were collected immediately after defecation for each individual and placed in a plastic container marked with the name, species, gender, and breeding base. Fecal samples were then stored at 4°C for DNA extraction. The detailed information of the samples is shown in Table 1.

**Table 1:** Non-human primate (NHP) subjects sampled in the study.

**Breeding bases**	**Species of NHPs**																			
	***Hylobates hoolock***	***Hylobates leucogenys***	***Nomascus gabriellae***	***Rhinopithecus*** **sp.**	***Macaca mulatta***	***Callithrix jacchus***	***Trachypithecus francoisi***	***Macaca assamensis***	***Erythrocebus patas***	***Macaca fascicularis***	***Colobus guereza***	***Eulemur fulvus***	***Papio*** **sp.**	***Mandrillus sphinx***	***Pan troglodytes***	***Pongo pygmaeus***	***Sapajus apella***	***Lemur catta***	***Saimiri sciureus***	**No. of samples**
Jing'an	5	0	11	11	0	0	5	4	6	1	8	5	2	4	0	0	2	0	0	64
Headquarter	0	2	7	5	0	4	0	0	0	0	0	0	0	0	5	3	0	0	0	26
Gaochun	0	0	10	3	0	0	0	0	0	0	0	0	0	0	0	0	6	5	2	26
Rescue center	0	0	0	0	4	0	0	0	0	0	0	0	0	0	0	0	0	0	0	4
No. of samples	5	2	28	19	4	4	5	4	6	1	8	5	2	4	5	3	8	5	2	120

### Detection of the Samples by Polymerase Chain Reaction

From each sample, 0.2 *g* of stool were used to isolate genomic DNA using the Stool DNA Kit (Tiangen Biotech, Beijing, China) according to the manufacturer's instructions. The genomic DNA was then detected by PCR assays based on the 16S or 18S rDNA sequence of six *Entamoeba* species according to previous studies (15–18). *E. chattoni* and *E. nuttalli* were detected by one round PCR, while *E. histolytica, E. dispar, E. moshkovskii*, and *E. coli* were detected by nested PCR using specific primers (Table 2). After the final PCR reaction, the products were examined by agarose gel electrophoresis, and the size of target fragments was validated by comparing them with DL2000 DNA Marker (TaKaRa, Dalian, China).

**Table 2:** Primers used for *Entamoeba* spp. detection.

**Genus/species**	**Primers (5'−3')**	**PCR product size (bp)**	**Reference**	**Gene**
*Entamoeba* genus	E1-F: TAAGATGCAGAGCGAAA	*E. histolytica*: 897	(15)	16S-rDNA
	E1-R: GTACAAAGGGCAGGGACGTA	*E. dispar*: 898		
		*E. moshkovskii*: 896		
*E. histolytica*	EH-F: AAGCATTGTTTC TAGATCTGAG	439		
	EH-R: AAGAGGTCTAACCGAAATTAG			
*E. dispar*	ED-F: TCTAATTTCGATTAGAACTCT	174		
	ED-R: TCCCTACCTATTAGACATAGC			
*E. moshkovskii*	EM-F: GAAACCAAGAGTTTCACAAC	553		
	EM-R: CAATATAAGGCTTGGATGAT			
*Entamoeba* genus	Entam1: GTTGATCCTGCCAGTATTATATG	631	(16)	16S-rDNA
	Entam2: CTTTAAGTTTCAGCCTTGTGACC			
*E. coli*	EC-F: GAATGTCAAAGCTAATACTTGACG	159	(17)	16S-rDNA
	EC-R: GATTTCTACAATTCTCTTGGCATA			
*E. chattoni*	ECH-F: AGGATTTGTTTTATAACAAGTTC	215	(16)	16S-rDNA
	ECH-R: TAAATAACCTTTCTCCTTTTTCTATC			
*E. nuttalli*	EN-F: TTTTAACATTTTGAAGACTTTGATA	449	(18)	18S-rDNA
	EN-R: AAGGTAATATTGATATACTCAGATTA			

### Sequence Analysis and Data Accessibility

Positive samples with the correct molecular size were purified and confirmed by two-directional sequencing using the specific PCR primers for each species by Sangon Biotech (Shanghai, China). The obtained nucleotide sequences were aligned with published international reference sequences available from the NCBI GenBank™ database using BLAST.

The assembled sequences obtained in this study were submitted to GenBank under accession number ON254797 for *E. histolytica* (9 samples), ON254798 (1 sample) and ON254799 (1 sample) for *E. nuttalli*, ON254800 for *E. chattoni* (12 samples), ON254801 (21 samples) and ON254802 (6 samples) for *E. coli*, and ON254803 for *E. dispar* (27 samples).

### Statistical Analysis

The samples with the sequence that can be assigned to the reference sequences were defined as positive, and used to calculate the prevalence of *Entamoeba* spp. infections. The statistical analysis was performed to analyze the risk factors, including breeding bases and genders, for *Entamoeba* spp. infections in NHPs using SPSS 25.0. A chi-square test was used to analyze the non-metric variables in the form of frequency tables. *P* < 0.05 was considered significant.

## Result

### *Entamoeba* spp. Infection in NHPs

Among 120 stool samples from NHPs, 59 (49.17%) were detected as *Entamoeba* spp. positive. As shown in Table 3, the most common species detected were *E. coli* and *E. dispar* in 27 (22.50%) samples, respectively, followed by *E. chattoni* (10.00%, 12/120), *E. histolytica* (7.50%, 9/120) and *E. nuttalli* (1.67%, 2/120). None sample presented infection with *E. moshkovskii*. Statistical analysis showed that there was a significant difference in the infection rate of different *Entamoeba* spp. in the NHPs (*X*^2^ = 63.003, *p* = 0.000).

**Table 3:** Mono- and mixed infection of *Entamoeba* spp. in NHPs in Nanjing Hongshan Forest Zoo.

**Species of** ***Entamoeba***	**Species of NHPs**																			**No. (%) of samples**
	***Hylobates hoolock***	***Hylobates leucogenys***	***Nomascus gabriellae***	***Rhinopithecus*** **sp.**	***Macaca mulatta***	***Callithrix jacchus***	***Trachypithecus francoisi***	***Macaca assamensis***	***Erythrocebus patas***	***Macaca fascicularis***	***Colobus guereza***	***Eulemur fulvus***	***Papio*** **sp.**	***Mandrillus sphinx***	***Pan troglodytes***	***Pongo pygmaeus***	***Sapajus apella***	***Lemur catta***	***Saimiri sciureus***	
**Species in monoinfections**																				42(35.00)
*E. histolytica*	0	0	3	1	0	0	0	0	0	0	0	0	0	0	0	0	0	0	0	4(3.33)
*E. dispar*	0	0	1	3	0	2	0	0	1	0	5	1	0	0	1	0	1	1	0	16(13.33)
*E. moshkovskii*	0	0	0	0	0	0	0	0	0	0	0	0	0	0	0	0	0	0	0	0
*E. coli*	2	0	3	0	0	0	3	0	0	0	0	0	0	0	1	1	0	2	0	12(10.00)
*E. chattoni*	1	1	1	1	1	0	0	1	2	1	0	0	0	0	0	0	0	0	0	9(7.50)
*E. nuttalli*	0	0	0	0	0	0	0	0	0	0	0	0	0	0	1	0	0	0	0	1(0.83)
**Species in mixed infections**																				17(14.17)
*E. dispar+ E. chattoni*	1	0	0	0	0	0	0	0	0	0	0	0	0	0	0	0	0	0	0	1(0.83)
*E. coli+ E. dispar*	0	0	4	0	0	0	1	1	0	0	0	0	0	0	0	2	1	0	0	9(7.50)
*E. coli+ E. histolytica*	0	0	1	1	2	0	0	0	0	0	0	0	0	0	0	0	0	0	0	4(3.33)
*E. chattoni+ E. histolytica*	0	0	0	0	1	0	0	0	0	0	0	0	0	0	0	0	0	0	0	1(0.83)
*E. coli+ E. nuttalli*	0	0	0	0	0	0	0	0	0	0	0	0	0	0	1	0	0	0	0	1(0.83)
*E. coli+ E. dispar+ E. chattoni*	0	0	0	0	0	0	0	0	1	0	0	0	0	0	0	0	0	0	0	1(0.83)

### Multiple Infections

Among the 59 positive stool samples, 17 presented multiple infections (14.17%), from which 16 corresponded to co-infections and 1 to triple infection. Co-infections *E. coli* + *E. dispar* represented 52.94% (9/17) of the co-infections, and the only case of triple infection was with *E. coli* + *E. dispar* + *E. chattoni* (Table 3).

Multiple infections were detected in 9 among the 19 species of NHPs. The highest rate of multiple infection was revealed in *M. mulatta* (75.0%, 3/4), followed by *P. pygmaeus* (66.7%, 2/3). The multiple infections of the other species are shown in Table 3.

### Risk Factors for the Infection of *Entamoeba*. spp in NHPs

From the 19 species of NHPs, *M. mulatta, M. fascicularis*, and *P. pygmaeus* were all positive for *Entamoeba* spp. infections, while none of the fecal samples obtained from *Papio* sp., *M. sphinx*, and *S. sciureus* were detected as positive. The infection rates of other species of NHPs are shown in Table 4.

**Table 4:** Factors associated with prevalence of *Entamoeba* spp. in NHPS in this study.

**Variable**	**No. of positive/tested**	**Prevalence (%)**	***X*** **^2^**	***P*** **value**
**Species**			*	*
*Hylobates hoolock*	4/5	80.00		
*Hylobates leucogenys*	1/2	50.00		
*Nomascus gabriellae*	13/28	46.42		
*Rhinopithecus* sp.	6/19	31.58		
*Macaca mulatta*	4/4	100.0		
*Callithrix jacchus*	2/4	50.00		
*Trachypithecus francois*	4/5	80.00		
*Macaca assamensis*	2/4	50.00		
*Erythrocebus patas*	4/6	66.67		
*Macaca fascicularis*	1/1	100.0		
*Colobus guereza*	5/8	62.50		
*Eulemur fulvus*	1/5	20.00		
*Papio* sp.	0/2	0		
*Mandrillus sphinx*	0/4	0		
*Pan troglodytes*	4/5	80.00		
*Pongo pygmaeus*	3/3	100.0		
*Sapajus apella*	2/8	25.00		
*Lemur catta*	3/5	60.00		
*Saimiri sciureus*	0/2	0		
**Breeding base**			4.987	0.173
Jing'an	30/64	46.88		
Headquarter	14/26	53.85		
Gaochun	11/26	42.30		
Rescue center	4/4	100.0		
**Gender**			1.420	0.492
Male	18/39	46.15		
Female	25/54	46.30		
Unknown	16/27	59.26		

* *Considering the small sample size of each species of NHPs, significance test was not analyzed among the species of NHPs*.

As shown in Table 4, all the samples from the rescue center presented *Entamoeba* infection, while the infection rates of the other three breeding bases were close to 50.0%. No statistical significance was observed among different bases (*X*^2^ = 4.987, *p* = 0.173).

To assess the effect of NHPs gender on *Entamoeba* spp. infection, the infection rates of the gender categories (male, female, and unknown) were analyzed. It should be noted that there was no significant association of *Entamoeba* spp. infection with gender (*X*^2^ = 1.420, *p* = 0.492) (Table 4). Nevertheless, no *E. histolytica-*positive samples were detected in the NHPs male category.

## Discussion

The *Entamoeba* genus contains many species residing in the intestinal lumen, of which *E. histolytica* is the main species associated with pathological sequelae (9). Nevertheless, other species are also frequently detected in a range of animals, such as goat, pig, yak, mice, and alpacas (7, 19–22). In addition, both captive and free-ranging NHPs have been identified as common hosts of *Entamoeba* spp. (6, 11, 14).

The present study provides the first information on the epidemiological data of *Entamoeba* spp. infections in NHPs from Nanjing Hongshan Forest Zoo (49.17%, 59/120). The prevalence rate of *Entamoeba* spp. was lower than in the free-ranging NHPs in savanna woodland (Tanzania, *Pan troglodytes*, 79%) (23) and Taihangshan (China, *Macaca mulatta tcheliensis*, 89.96%) (11). Compared to zoological gardens housing captive NHPs, the positivity was higher than those in Ibadan (Nigeria, *Erythrocebus patas, Cercocebus atys, Mandrillus sphinx, Cercopithecus sabaeus*, 13.90%) (14), Belgium (Belgium, prosimians, New World monkeys, Old World monkeys and apes, 44%) (24), and the experimental macaques in Yunnan (China, *M. mulatta, M. nemestrina and M. fascicularis*, 9.31%) (13). These results indicate that free-ranging NHPs may have a much higher *Entamoeba* occurrence rate than captive ones. It is likely that captive NHPs are largely housed in zoological gardens and research facilities, and the captive management may interrupt the transmission of parasitic pathogens to hosts. Of note, the infection rate of *Entamoeba* spp. in captive NHPs from Nanjing Hongshan Forest Zoo is much higher than those reported in several epidemiological studies (seven NHPs species) (13, 14) suggesting that this attraction should strengthen the housing and husbandry practices to improve the health of NHPs.

Except for *E. moshkovskii*, the other five *Entamoeba* spp. species were found in this study, presenting different infection rates. Compared with the studies in Belgium and the Netherlands (four *Entamoeba* species among 36 NHPs species), China (three *Entamoeba* species among three NHPs species) and Italy (three *Entamoeba* species among nine NHPs species), more species of *Entamoeba* spp. were detected in Nanjing Hongshan Forest Zoo (6, 13, 25). The infection rate of *E. histolytica* was 7.50%, which is consistent with the data reported by the zoological gardens in Belgium and Netherlands (36 NHPs species, 8.1%) and the experimental macaques (*M. mulatta, M. nemestrina and M. fascicularis*, 6.38%) in Yunnan Province in China (6, 13). In contrast, no *E. histolytica* infection was detected in savanna woodland (Tanzania, *Pan troglodytes*), Taihangshan (China, *Macaca mulatta tcheliensis*), and an Italian zoological garden (Italy, nine NHP species) (11, 23, 25). Our results revealed higher diversity of *Entamoeba* species in NHPs in Nanjing Hongshan Forest Zoo China, and these NHPs may be potential sources for human infection with *E. histolytica*.

*Entamoeba dispar* and *E. coli* were the more prevalent species revealed in this study, consistent with other 13 zoos/parks in China (26). While in the NHPs from zoological gardens in the UK and the experimental NHPs from Japan and China, only *E. dispar*/*E. coli* was detected as the main species for infections (27–29). In addition, *E. hartmanni* was the most prevalent *Entamoeba* species shed in the NHPs in Belgium and Netherlands, and in savanna woodland chimpanzees (6, 23), and *E. chattoni* was the predominant species shed in macaques from wild Taihangshan and southwest China (11, 30). These results indicated that different regions had their specific prevalent species of *Entamoeba*, which might be related to the parameters including lifestyles, and the species of NHPs. The observation needs to be further studied. There is, to date, no report indicating the *E. moshkovskii* infections in NHPs (11, 25, 31). Likewise, no *E. moshkovskii*-positive sample was detected in the NHPs in this study. However, the cases of human infection with *E. moshkovskii* have been reported in Malaysia, Japan and Colombia (32–34), and *E. moshkovskii* has also been detected in animal hosts, such as cattles (UK), elephants (Namibia), and chelonian (Mexico) (35, 36). Further investigations are needed to demonstrate whether NHPs can be infected by *E. moshkovskii*.

The multiple infection rate of *Entamoeba* spp. in the NHPs in this study was 14.17%. It was lower than that in the other 13 zoos/parks in China (45.4%) and in the free-ranging Taihangshan Macaques (84.93%) (11, 25). The differences in the prevalence of multiple infections may be related to the method used for *Entamoeba* detection. Microscopic examination was used in those studies, which may not allow correct discriminations of *Entamoeba* species. Compared with the studies using molecular techniques, the multiple infection rate in this study was consistent with the studies in Tanzania (16.67%) (22), higher than that in Italian (0) (24), and lower than that in Belgium and the Netherlands (51.90%) (6, 22, 24). The difference in *Entamoeba* multiple infections in NHPs may be associated with the climate, the species of NHPs or breeding and management conditions, which need to be further studied. *Entamoeba* spp. was prevalent in all four breeding bases of the zoo, especially in the rescue center. It could attribute to the poor conditions in which the NHPs were raised before being rescued. Importantly, both males and females have a high infection rate, whereas no significant difference was observed (*X*^2^ = 1.42, *p* = 0.492), which was consistent with the finding in the experimental macaques in Yunnan (13). It indicates that the infection of *Entamoeba* spp. was not correlated to the gender of NHPs. However, all the *E. histolytica*-positive samples were detected from females, which warrants further investigations.

In the 19 species of NHPs, no *M. sphinx* and *Papio* sp. was confirmed with *Entamoeba* spp. infections. In a previous study, both the semi-free-ranging (100%) and captive (81.20%) *M. sphinx* presented a high infection rate of *Entamoeba* spp. (26, 37), and *Papio* sp. was verified to be a natural host of *Entamoeba* spp. (38). It is likely that our sampling size was too small to identify the positive infection of *Entamoeba* spp. in these two NHPs species. In Martinez et al.'s research, *S. sciureus* was not susceptible to *E. histolytica* (39). In our study, *S. sciureus* was found to be *Entamoeba* spp. negative, which could be explained by the small sample size. The infection rate of *Entamoeba* spp. ranged from 25% (*Sapajus apella*) to 100% (*M. mulatta, Macaca fascicularis*, and *P. pygmaeus*), indicating that the infection of *Entamoeba* spp. was widespread in most species of NHPs in this zoo.

In conclusion, this study revealed that *Entamoeba* spp. are widely prevalent in the captive NHPs in Nanjing Hongshan Forest Zoo. Furthermore, five species (*E. coli, E. dispar, E. histolytica, E. chattoni*, and *E. nuttalli*) were identified, with *E. coli* and *E. dispar* as the predominant species. The epidemiology of *Entamoeba* spp. infection is complex, and these captive NHPs could potentially participate in zoonotic transmission, and the *Entamoeba* spp. infection of other animals from this zoological garden. The prevention and control of *Entamoeba* spp. infection in this zoological garden should be strengthened to reduce disease exposure risk of public health.

## Data Availability Statement

The datasets supporting the findings of this article are included within the article. Additional datasets generated for this study are available on reasonable request to the corresponding authors.

## Ethics Statement

The animal study was reviewed and approved by the Animal Care and Welfare Committee of Anhui Science and Technology University, Fengyang, China. Written informed consent was obtained from the owners for the participation of their animals in this study.

## Author Contributions

XL designed this study, performed data analysis, and drafted the manuscript. GB, MY, YYG, and YF helped to carry out the study. WL and YFG helped to design this study and perform data analysis. WC participated in the collection of experimental materials. ML critically revised the manuscript. All authors read and approved the final version of the manuscript.

## Funding

This work was supported by the Modern Cattle and Goat Industrial Technology System Program of Anhui Province, the Anhui University Collaborative Innovation Project (GXXT-2019-035), the Anhui Provincial Natural Science Foundation (1808085MC84 and 1908085QC116), the School-Level Talent Introduction Project of Anhui Science and Technology University (DKYJ201902), the Natural Science Research Projects of Anhui Science and Technology University (2021lzryb08), and National College Students Innovative and Entrepreneurial Training Program (202110879064 and 202010879040).

## Conflict of Interest

The authors declare that the research was conducted in the absence of any commercial or financial relationships that could be construed as a potential conflict of interest.

## Publisher's Note

All claims expressed in this article are solely those of the authors and do not necessarily represent those of their affiliated organizations, or those of the publisher, the editors and the reviewers. Any product that may be evaluated in this article, or claim that may be made by its manufacturer, is not guaranteed or endorsed by the publisher.
